# Ending the Pandemic: Are Rapid COVID-19 Tests a Step Forward or Back?

**DOI:** 10.5811/westjem.2021.2.50550

**Published:** 2021-05-19

**Authors:** Tony Zitek, Joseph B. Fraiman

**Affiliations:** * Herbert Wertheim College of Medicine, Florida International University, Department of Emergency Medicine, Miami, Florida; † Lallie Kemp Regional Medical Center, Department of Emergency Medicine, Independence, Louisiana

## Abstract

Some experts have promoted the use of rapid testing for COVID-19. However, with the current technologies available, continuing to replace laboratory-based, real-time reverse transcription polymerase chain reaction tests with rapid (point-of-care) tests may lead to an increased number of false negative tests. Moreover, the more rapid dissemination of false negative results that can occur with the use of rapid tests for COVID-19 may lead to increased spread of the novel coronavirus if patients do not understand the concept of false negative tests. One means of combatting this would be to tell patients who have a “negative” rapid COVID-19 test that their test result was “indeterminate.”

Recent scholarly articles^1^ and popular media articles^2^ have pushed for increased availability of rapid (point-of-care) testing for coronavirus disease 2019 (COVID-19). There would indeed be many benefits to having an instantaneous means of accurately determining who has COVID-19 and who does not. However, with our current technologies and our current approach to diagnostic testing, we believe that increasing the use of rapid tests may be harmful as these tests will speed the dissemination of false negative results.

The criterion standard test for COVID-19 is a (non-rapid) laboratory-based, real-time reverse transcription polymerase chain reaction (rRT-PCR) test,^3^ which is generally performed as a nasal swab and takes at least 24 hours for results. The sensitivity of these rRT-PCR tests are about 70% (60–78%), but they have very high specificity.^4^ Thus, the issue with rRT-PCR tests for COVID-19 is the substantial false negative rate, which may be even higher if the test is performed by an oral swab rather than a nasal swab.^3^ False positive tests are less common but may occur from contamination of the specimen or reagents.^4^ Additionally, some asymptomatic patients who have a positive rRT-PCR test may be harboring remnants of severe acute respiratory syndrome coronavirus 2 (SARS-CoV-2), but may not be contagious.^5^ These patients (who are neither symptomatic nor contagious) should be considered to have a clinical false positive. No published data have reported how often this happens, but for the purposes of the ensuing calculations, we will assume the specificity of laboratory-based rRT-PCR swab tests is 99.5%.

With regard to rapid tests, there are two types: antigen and molecular. Antigen tests detect a viral protein, and molecular tests detect viral RNA. A recent systematic review estimated that when using the rRT-PCR tests as the criterion standard, the rapid antigen tests have a sensitivity of 56.2% (95% confidence interval [CI], 29.5–79.8%) and a specificity of 99.5% (95% CI, 98.1–99.9%) while rapid molecular tests have a sensitivity of 95.2% (95% CI, 86.7–98.3%) and specificity 98.9% (95% CI, 97.3–99.5%).^6^ Among the molecular tests that were assessed, the Xpert Xpress assay (Cepheid Inc., Sunnyvale, CA) appears to have the highest sensitivity at 99.4% (95% CI, 98.0–99.8%), which is substantially higher than the commonly used ID NOW (Abbott Laboratories, Chicago, IL), which has a sensitivity of 76.8% (95% CI, 72.9–80.3%). However, the specificity of Xpert Xpress appears to be a little lower than that of ID NOW at 96.8% (95% CI, 90.6–99.0%) as compared to 99.6% (95% CI, 98.4–99.9%).^6^

When interpreting these data, it is important to emphasize that the criterion standard (rRT-PCR) used for these calculations also has moderate sensitivity (around 70%) and imperfect specificity (around 99.5%). Therefore, if the sensitivity and specificity of a rapid antigen test are 56% and 99.5%, respectively, compared to rRT-PCR, we would expect the overall sensitivity to be about 39% and the overall specificity to be about 99%. Thus, if 1000 people had a rapid antigen test, and 100 (10%) of them truly had COVID-19, we would expect the following:

-39 of the 100 patients with COVID-19 would be identified with this test (with a true positive result);
-48 patients would test positive for COVID-19, but 9 (18.8%) of those would be false positive results;
- 952 patients would test negative for COVID-19, but 61 (6.4%) of those would be false negative results.

Thus, in the above scenario and detailed in Table 1, nearly 1 in 5 (18.8%) positive tests represents a false positive, and even more concerning, the majority of patients (61%) with COVID-19 (n= 100 by design in the example) would have a negative test (61 false negatives + 39 true negatives = 100 infections). Consequently, we are concerned that without substantial proviso, rapid antigen tests lack sufficient accuracy to be used clinically. In particular, we are concerned about the potential widespread use of the rapid antigen test made by Abbott Laboratories. This test, which is reported to only cost $5, recently gathered attention in the popular media after receiving emergency use status from the US Food and Drug Administration.^7^ While there are currently insufficient data to precisely report this particular test’s sensitivity and specificity, it is likely similar to the average for antigen tests mentioned above. However, even if a rapid test that has a sensitivity similar to that of rRT-PCR is used, we still believe that replacing the laboratory-based rRT-PCR tests with rapid tests could be harmful, as rapid tests will likely increase the spread of SARS-CoV-2 in patients with false negative results.

Until recently, the impact of false negative COVID-19 tests has likely been dampened both by government-mandated closures and prolonged wait times for rRT-PCR test results. With many businesses and schools closed, a patient with a false negative COVID-19 test had less ability to widely spread SARS-CoV-2. Additionally, days-long delays in access to results have been frustrating to patients and physicians. However, patients’ isolation behavior is likely stricter during the waiting period for results than following a negative result. Because the period of preventable transmission for SARS-CoV-2 (typically less than 10 days following initiation of symptoms for mild to moderate COVID-19) is coincident with most waiting periods for results, it seems likely that the delay in results has offered some measure of unrecognized protection. This means that with the increasing use of rapid COVID-19 tests, transmission of SARS-CoV-2 could actually increase. More immediate results mean more immediate false “negatives” and, likely, less concerted self-isolation behavior.

The idea that the wait time for the results of COVID-19 tests is protective is supported by one survey study that asked respondents to describe their isolation behaviors during a hypothetical outbreak involving a potentially fatal contagious respiratory illness. Respondents who were uncertain of their own transmissibility to vulnerable individuals reported they would engage in social isolation behaviors at the same rate that they reported for scenarios in which they knew they would infect vulnerable individuals.^8^ Therefore, the wait period for the results of an rRT-PCR test likely produces a healthy uncertainty that is more associated with appropriate isolation behavior than the behavior after a (potentially false) negative test result.

We believe the main reason for this is that most patients and some healthcare professionals do not understand the concept of false negative tests. We fear that patients who have false negative tests may immediately return to work or school or get on a plane, even if symptomatic and even if counseled to stay home until symptoms resolve. Worse, some healthcare professionals may not fully understand how to incorporate the sensitivity of a diagnostic test into their decision-making and may not provide appropriate counsel to patients with negative test results. Indeed, previous work has demonstrated that both physicians and patients have trouble interpreting and applying healthcare statistics.^9^

Even if all physicians knew how to appropriately counsel patients with negative COVID-19 tests, the current testing strategy for COVID-19 often bypasses physician assessment of the patient, compounding the problem of false negative tests. Medical tests have traditionally been ordered and interpreted by physicians. However, tests for COVID-19 can now be done at a drugstore, in a drive-thru testing site, or at home without a healthcare professional’s involvement. While increased access to testing is a good thing, misinterpretation of test results is dangerous. Many tests for COVID-19 are being done without any consideration for the pretest probability, without which we cannot properly assess the results. Most people who get the test just assume that a positive test means they have COVID-19, while a negative test means they do not. With no healthcare professional to counsel the patient, the patient will not know any better.

Now, reconsider the scenario above where 1000 rapid antigen tests were performed on a population where 10% actually had COVID-19. In an ideal world, all of the 61 patients who had false negative results would remain in quarantine despite their negative test results, and they would not spread SARS-CoV-2 to anyone new. However, suppose 20% (12 patients) of those 61 patients with a false negative test return to work, school, or social situations early because they believe they do not have COVID-19. If six of these 12 patients spreads SARS-CoV-2 to just one new person that would not have been exposed to the virus had the patient remained in quarantine for 2–3 more days, then the transition from rRT-PCR tests to rapid antigen tests accounts for six new cases of COVID-19 in this group of 1000 tested patients. Over hundreds of thousands or millions of rapid antigen tests, the increased spread of SARS-CoV-2 through this mechanism could be striking.

At this point, rapid testing for COVID-19 is already widely used. The lack of accuracy of the tests seems to be less important to some decisionmakers than the fact that they give a result quickly. In a few special circumstances where repeated tests for COVID-19 are performed on asymptomatic individuals, such as testing done by the National Football League, rapid testing may be preferred to laboratory-based testing for logistical reasons. However, in typical healthcare settings, rapid tests are not optimal and must be used cautiously. Therefore, as a means to reduce the spreading of SARS-CoV-2 from patients who have had “negative” rapid COVID-19 tests, we recommend two potential solutions. First, negative results of rapid tests should be called “indeterminate.” This is in fact more accurate than saying the test was negative (since possibly over 50% of patients with COVID-19 will have a false negative). For outpatients with possible COVID-19, this would serve as a constant reminder that they should remain in self-isolation until their symptoms resolve even if their test is negative.

Second, in cases where patients are being admitted to the hospital with a clinical presentation suggestive of COVID-19, the patient should continue to be isolated even if they have a negative rapid test. For these patients, additional testing for COVID-19 should be considered, and for some of these patients, a computed tomography (CT) of the chest should be performed. Notably, some society guidelines recommend against the use of CT of the chest for the diagnosis of COVID-19, and the harms of CT with regard to radiation and cost are important to consider.^10^, ^11^ However, the combination of rRT-PCR and CT provides a very high sensitivity (about 97%) for COVID-19^4^; thus, the selective use of this strategy could reduce the potential harms of false negative tests discussed above.

In summary, regardless of the type of test used for virologic confirmation of SARS-CoV-2, there are a substantial number of false negative tests. Rapid tests, especially rapid antigen tests, likely have even higher numbers of false negatives. Therefore, policies for quarantine and isolation that rely solely on the results of a rapid test are bound to result in misdiagnosis and increased viral transmission. Informing patients that their rapid COVID-19 test was negative could result in less self-isolation and increased viral spread. If rapid tests *are* used, we recommend that negative results instead be called “indeterminate” to remind patients that a negative test does not mean they do not have COVID-19.

## Figures and Tables

**Table 1:** The hypothetical results of 1000 rapid antigen tests for COVID-19 in a group with a 10% disease prevalence.

Diseased	Non-diseased	
True Positives = 39	False Positives = 9	Total Positives = 48
False Negatives = 61	True Negatives = 891	Total Negatives = 952
